# Expression of microRNAs in leukocytes and serum of asbestosis patients

**DOI:** 10.1186/s40001-023-01129-z

**Published:** 2023-05-15

**Authors:** Vivien Kauschke, Monika Philipp-Gehlhaar, Joachim Schneider

**Affiliations:** Institute and Outpatient Clinic of Occupational and Social Medicine, University Hospital of Giessen and Marburg, Aulweg 129, 35392 Giessen, Germany

**Keywords:** MicroRNA, Leukocytes, Serum, Pleural plaques, Asbestosis

## Abstract

**Background:**

Although asbestos use is banned in many countries, long latency of asbestos-related diseases like pleural plaques or asbestosis mean it is still a public health issue. People suffering from these diseases have a higher risk of developing mesothelioma or lung cancer, which can progress quickly and aggressively. MicroRNAs were suggested as potential biomarkers in several diseases. However, in asbestosis, blood microRNAs are less explored. Since miR-32-5p, miR-143-3p, miR-145-5p, miR-146b-5p, miR-204-5p and miR-451a are involved in fibrotic processes and in cancer, expression of these microRNAs was analyzed in leukocytes and serum of asbestosis patients.

**Methods:**

MicroRNA expression was analyzed in leukocytes and serum of 36 patients (26 affected by pleural plaques and 10 by asbestosis) and 15 healthy controls by real-time RT-PCR. Additionally, data analyses were performed regarding disease severity based on ILO classification.

**Results:**

MicroRNA miR-146b-5p was significantly down-regulated in leukocytes of patients suffering from pleural plaques with a large effect indicated by *η*^2^_p_ = 0.150 and Cohen’s *f* = 0.42, a value of difference of 0.725 and a 95% confidence interval of 0.070–1.381. In patients suffering from asbestosis miR-146b-5p was not significantly regulated. However, data analyses considering disease severity only, revealed that miR-146b-5p was significantly down-regulated in leukocytes of mildly diseased patients compared to controls with a large effect indicated by *η*^2^_p_ = 0.178 and Cohen’s *f* = 0.465, a value of difference of 0.848 and a 95% confidence interval of 0.097–1.599. Receiver operating characteristic (ROC) curve and an area under the ROC curve value of 0.757 for miR-146b-5p indicated acceptable discrimination ability between patients suffering from pleural plaques and healthy controls. Less microRNAs were detectable in serum than in leukocytes, showing no significant expression differences in all participants of this study. Moreover, miR-145-5p was regulated significantly differently in leukocytes and serum. An *R*^2^ value of 0.004 for miR-145-5p indicated no correlation in microRNA expression between leukocytes and serum.

**Conclusion:**

Leukocytes seem more suitable than serum for microRNA analyses regarding disease and potentially cancer risk assessment of patients suffering from asbestos-related pleural plaques or asbestosis. Long-term studies may reveal whether down-regulation of miR-146b-5p in leukocytes might be an early indicator for an increased cancer risk.

## Background

Approximately 107,000 deaths occur due to asbestos-related diseases in Europe every year [1]. In the European Union (EU) 78% of occupational cancers were caused by asbestos exposure [2]. Although asbestos use has been prohibited in many countries, a long latency of 30–40 years after asbestos exposure [1] makes asbestos-related diseases still relevant today. Despite banning asbestos use in new buildings, the demolition of older buildings can release asbestos fibers and expose workers [1]. Today, around 4.1–7.3 million workers in the EU are exposed to asbestos, mostly people working in the construction industry [2]. Moreover, several countries permit the use of asbestos, potentially exposing approximately 125 million people worldwide [3–5].

Inhaled asbestos fibers can cause inflammation resulting in fibrosis of the pleura and/or the lung [6]. Pleural plaques are composed of avascular collagen fibers, which can calcify [7]. They can be seen as indicators of asbestos exposure and they are the most common condition in asbestos-exposed people [8].

Asbestosis is defined as fibrosis of lung interstitium following reliable asbestos exposure [9].

Although pleural plaques or asbestosis are considered as benign diseases [10], studies have shown that people affected by asbestos-related pleural plaques or asbestosis have a higher risk of developing mesothelioma or lung cancer [11, 12].

Inflammatory processes play a key role in the pathogenesis of occupational-related pneumoconiosis, such as asbestosis [13–18]. As a result, cell proliferation, cell transformation and tumor formation can be stimulated [19].

It has been shown that asbestos-related diseases can influence microRNA regulation in peripheral blood [20]. MicroRNAs are very short ~21–23 nucleotide long non-coding RNAs, which have raised interest as biomarkers for several diseases [21–24]. They are involved in important processes like proliferation, differentiation and apoptosis of cells through post-transcriptional regulation of gene expression [25]. By binding target mRNAs, microRNAs can induce repression of translation or mRNA degradation [5, 26]. MicroRNAs are expressed in many tissues and can be released into the circulation or extracellular compartment by damaged tissue [27, 28]. Since they are obtainable in body fluids, such as saliva, urine, blood, plasma or serum [29] microRNAs offer the advantage of a non- or less invasive approach of disease monitoring [22, 24]. In lupus nephritis urinary microRNAs helped to detect early fibrosis and to foresee progression of the disease [30]. Circulating microRNAs were suggested as potential biomarkers in diseases, such as B-cell lymphoma [31], lung and prostate cancer [32]. Besides, expression of circulating microRNAs supported the prediction of an early response to treatment in patients suffering from chronic myeloid leukemia [33, 34].

It has been shown for prostate cancer that tumors directly released microRNAs into plasma or serum [32]. Moreover, ovarian cancer produced a distinctive microRNA profile in peripheral blood cells [35], and in mesothelioma patients, miR-103 levels in the cellular fraction of whole blood were significantly down-regulated [20]. Thus, leukocytes- and/or serum-derived microRNAs might be suitable for evaluating the course of disease and possibly cancer risk. Several studies have identified biomarkers for the detection of asbestos-related cancer, usually in tissue [1, 5, 36]. However, less is known about the suitability of microRNAs in comparatively easily obtainable blood samples, as biomarkers to evaluate disease progression of patients affected by asbestos-related diseases, like pleural plaques or asbestosis. MicroRNA analysis in blood might be a useful and minimally invasive approach to assess a possible risk of developing malign asbestos-related cancer.

It is known that the microRNAs miR-32-5p, miR-143-3p, miR-145-5p, miR-146b-5p, miR-204-5p and miR-451a are involved in fibrosis [37–42] and cancer [36, 43–48]. Therefore, the aim of this study was to identify microRNAs expressed in leukocytes and serum of patients affected by asbestos-related pleural plaques or asbestosis, which are possibly suitable as biomarkers for monitoring the disease progression.

## Materials and methods

### Study participants

Participants of the underlying study comprised 36 male individuals with an average age of 73 years affected either by asbestos-related pleural plaques (J92.0; ICD-10-GM version 2022) or by asbestosis (J61 and J92.0; ICD-10-GM version 2022) and 15 healthy individuals who served as controls. The patient collective comprised male individuals since only men with occupational asbestos-related changes in pleura and/or lung presented to our clinic, which can be explained by the larger proportion of men working in the construction industry. The asbestosis group included patients who showed asbestos-related changes in both, pleura and lungs. Diagnosis was based on physical examination, pulmonary function testing, hematological and biochemical analyses of blood samples and chest X-ray radiography. X-rays of pleura and lungs were evaluated according to the International Classification of Occupational and Environmental Respiratory Diseases (ICOERD) and to the International Classification of Radiographs of Pneumoconiosis of the International Labour Organization (ILO) [49]. The latter is shown in Table 1.

**Table 1:** X-ray classification of pleural plaques and asbestosis according to ILO

Grade	Classification
Classification of pleural changes	
0	No definite pleural plaques
1	< ¼ of lateral thoracic wall
2	¼—½ of lateral thoracic wall
3	> ½ of lateral thoracic wall
Hyalinosis complicata	Costophrenic obliteration, diffuse pleural thickening and pleuro-parenchymal fibrous strand (“crow feet”)
Classification of parenchymal changes	
< 1/0	No definite lung fibrosis
1/1 and 1/2	Mild lung fibrosis (asbestosis)
2/1, 2/2 and 2/3	Moderate lung fibrosis (asbestosis)
3/2, 3/3 and 3/ +	Severe lung fibrosis (asbestosis)

According to the ILO or ICOERD, patients suffering from pleural plaques or asbestosis were classified into three groups considering the degree of severity (mild, moderate or severe).

The control group included 1 female and 14 male participants with an average age of 37 years who were not exposed to asbestos in the past and underwent a preventive medical check-up in our clinic.

The relatively young age of participants in the healthy control group compared to the group of patients affected by asbestos-related pleural plaques or by asbestosis, is based on the circumstance that the majority of older people who presented to our clinic, showed comorbidities (e.g., hypertension or type II diabetes mellitus). Unhealthy people were excluded from the control group. Besides, asbestos-related pleural plaques or asbestosis rarely emerges in younger people due to the long latency period. Hence, participants affected by asbestos-related pleural plaques or asbestosis were older compared to healthy control participants.

For serum microRNA analysis, whole blood was drawn from all 36 patients of whom 26 patients were affected by pleural plaques and 10 patients affected by asbestosis as well as from 15 healthy controls who were not exposed to asbestos. In reference to ICOERD and ILO, 21 of these patients (18 pleural plaques and 3 asbestosis) were mildly diseased (ILO: pleural changes 1 and/or parenchymal changes 1/1 and 1/2), 9 patients (5 pleural plaques and 4 asbestosis) were moderately diseased (ILO: pleural changes 2 and/or parenchymal changes 2/1, 2/2 and 2/3) and 6 patients (3 pleural plaques and 3 asbestosis) were severely diseased (ILO: pleural changes 3 and/or parenchymal changes 3/2, 3/3 and 3/+).

Leukocytes were obtained from the whole blood of 15 control individuals and from 34 out of 36 patients of whom 24 patients were affected by pleural plaques and 10 patients affected by asbestosis. Twenty of these patients (17 pleural plaques and 3 asbestosis) were classified as mildly diseased (ILO: pleural changes 1 and/or parenchymal changes 1/1 and 1/2), 8 patients (4 pleural plaques and 4 asbestosis) as moderately diseased (ILO: pleural changes 2 and/or parenchymal changes 2/1, 2/2 and 2/3) and 6 patients (3 pleural plaques and 3 asbestosis) as severely diseased (ILO: pleural changes 3 and/or parenchymal changes 3/2, 3/3 and 3/+).

Blood samples, which were analyzed for serum microRNA but not for leukocytes microRNA belonged to two patients with pleural plaques, one with mild disease and the other with moderate disease.

### Sampling of whole blood

Blood sampling was approved by the local ethics commission of the department of medicine at the Justus-Liebig-University of Giessen, Germany (103/05 and 75/06). Patients gave written consent for their blood analysis in the underlying study. In total 36 patients and 15 healthy individuals were included in the study.

Whole blood was drawn by venipuncture on the inside of the elbow.

Blood samples of all 36 patients who were affected by either pleural plaques or asbestosis and 15 control individuals were analyzed for serum microRNAs as well as blood of 34 patients and 15 controls for leukocytes microRNAs. For leukocytes microRNA analysis three milliliters (ml) of whole blood was collected in Sarstedt Monovettes containing ethylenediaminetetraacetic acid (EDTA, Sarstedt, Nürmbrecht, Germany).

For serum analysis, 7 ml of whole blood was collected in Sarstedt S-Monovette® serum tubes (Sarstedt) without EDTA.

All blood samples were processed within 30 minutes (min) after collection. They were centrifuged for 10 min at 1900×*g* and 4 °C. Subsequently, separated plasma or serum was transferred into the respective RNase-free micro tubes (Sarstedt). To ensure cell-free plasma or serum samples, samples were centrifuged for another 10 min at 16,000×*g* and 4 °C and supernatants transferred into fresh RNase-free micro tubes (Sarstedt) before being stored at −80 °C pending further processing. From plasma separated EDTA blood was processed immediately for RNA isolation from leukocytes. Plasma samples were not analyzed for the underlying study.

### RNA isolation from leukocytes

Prior to RNA isolation, erythrocytes in the cellular portion of blood obtained after centrifugation as described above, were lyzed using maximally 1.5 ml of blood cells and 10 ml of erythrocytes lysis buffer (Buffer EL, Qiagen, Hilden, Germany). The mixture was incubated in RNase-free reaction tubes for 15 min on ice and then centrifuged for 10 min at 400×*g* and 4 °C. Subsequently, supernatants were removed, 5 ml of erythrocyte lysis buffer added to the pellet, resuspended and the mixture centrifuged again for 10 min at 400×*g* and 4 °C. Supernatants were removed again, the cell pellet resuspended in 1 ml erythrocyte lysis buffer, samples bisected and 500 microliters (µl) each transferred into two RNase-free reaction tubes (Sarstedt). Another 500 µl of erythrocyte lysis buffer was added to each tube and cells centrifuged for 10 min at 400×*g* and 4 °C. Supernatants were removed, leukocytes homogenized in 700 µl Qiazol (Qiagen) and incubated for 5 min at room temperature. Then 140 µl of chloroform (Carl Roth, Karlsruhe, Germany) was added, the samples were vigorously shaken or vortexed for 15 seconds (s) and then incubated for 3 min at room temperature. The mixtures were centrifuged for 15 min at 12000×*g* and 4 °C. Clear upper phase was transferred into a fresh RNase-free reaction tube (Sarstedt), 1.5 volumes of 100% ethanol added and RNA isolated using the miRNeasy Mini Kit (Qiagen) according to the manufacturer’s protocol. Isolated RNA was eluted in 30 µl RNase-free water and stored at −80 °C pending further processing.

### RNA isolation from serum

RNA was isolated from 100 µl of blood serum. Serum was mixed with 500 µl Qiazol (Qiagen) and incubated for 5 min at room temperature. After homogenization of serum samples, 3.5 µl Ce-miR-39_1 Spike-In Control (Qiagen) diluted to 1.6 × 10^8^ in RNase-free water containing 10 ng/µl carrier RNA from bacteriophage MS2 (Roche) was added to serum lysates to monitor microRNA recovery and reverse transcription efficiency in the following steps. Subsequently, 100 µl of chloroform was added and the samples were vigorously shaken or vortexed for 15 s and incubated for 2–3 min at room temperature. Afterwards, samples were centrifuged for 15 min at 12000×*g* and 4 °C. Clear upper phase was transferred into a fresh RNase-free reaction tube (Sarstedt) and 1.5 volumes of 100% ethanol added before RNA was isolated using the RNeasy Mini Elute columns and the miRNeasy Serum/Plasma Kit (Qiagen) according to manufacturer’s protocol.

### Reverse transcription of RNA from leukocytes and serum

cDNA was synthesized by reverse transcription of 250 ng of purified RNA from leukocytes or 1.5 µl of purified RNA from serum containing Ce-miR-39_1 Spike-In Control. RNA samples were reverse transcribed into cDNA for 24 hours at 37 °C using the miScript II RT Kit (Qiagen) with HiSpec buffer (Qiagen) and a cycler (Mastercycler gradient, Eppendorf, Hamburg, Germany). Volumes of components used for the reverse transcription reaction mix corresponded to manufacturer’s instructions. Two-hundred µl of RNase-free water were added to cDNA samples and microRNA expression was analyzed using real-time reverse transcription (RT)-polymerase chain reaction (PCR).

### Pre-screening for microRNAs expressed in leukocytes and serum using PCR Array

The miScript miRNA PCR Array Human Fibrosis (MIHS-117Z, Qiagen), which comprised 84 microRNAs expressed in fibrosis and additional normalization controls, reverse transcription controls as well as positive PCR controls, was used to pre-screen for microRNAs expressed in leukocyte or serum samples of two patients affected by moderate or severe pleural plaques, respectively. The miScript miRNA PCR Array Human Fibrosis was placed in a C1000 Touch™ Thermal Cycler with a CFX96 Real-time PCR Detection System (Bio-Rad, Munich, Germany) and cycling conducted according to manufacturer’s protocol as described below.

### Real-time reverse transcription polymerase chain reaction (real-time RT-PCR) in leukocytes and serum

After pre-screening for microRNAs expressed, real-time RT-PCR analyses of all patient samples were performed with the miScript SYBR Green PCR Kit (Qiagen) and miScript Primer Assays (Qiagen) in a Lightcycler System (Roche Diagnostics, Mannheim, Germany). MicroRNAs analyzed in leukocytes and serum were hsa-miR-32-5p (Accession No. MIMAT0000090), hsa-miR-143-3p (Accession No. MIMAT0000435), hsa-miR-145-5p (Accession No. MIMAT0000437), hsa-miR-146b-5p (Accession No. MIMAT0002809), hsa-miR-204-5p (Accession No. MIMAT0000265), and hsa-miR-451a (Accession No. MIMAT0001631). Volumes of components used for the real-time RT-PCR reaction mix corresponded to manufacturer’s instructions.

The cycling procedure started with an initial activation step at 95 °C for 15 min, followed by 40 cycles of denaturation at 94 °C for 15 s, annealing at 55 °C for 30 s and extension at 70 °C for 30 s. A melting curve was performed to check the purity of RT-PCR products. Negative controls received water instead of cDNA. For evaluation of microRNA expression in leukocytes or serum, delta cycle threshold (CT) values were analyzed using the pseudogene hsa-RNU6-6P as reference (Accession No. NG_034215) or the Spike-In Control Ce-miR-39_1 (Accession No. MIMAT0000010), respectively.

Table 2 summarizes all primers used for real-time RT-PCR.

**Table 2:** Human primers used for real-time RT-PCR

Primer	Sequence	Accession no.
hsa-miR-32-5p	UAUUGCACAUUACUAAGUUGCA	MIMAT0000090
hsa-miR-143-3p	UGAGAUGAAGCACUGUAGCUC	MIMAT0000435
hsa-miR-145-5p	GUCCAGUUUUCCCAGGAAUCCCU	MIMAT0000437
hsa-miR-146b-5p	UGAGAACUGAAUUCCAUAGGCUG	MIMAT0002809
hsa-miR-204-5p	UUCCCUUUGUCAUCCUAUGCCU	MIMAT0000265
hsa-miR-451a	AAACCGUUACCAUUACUGAGUU	MIMAT0001631
hsa-RNU6-6P	Sequence below table*	NG_034215
Ce-miR-39_1	UCACCGGGUGUAAAUCAGCUUG	MIMAT0000010

*sequence for hsa-RNU6-6P (U6 small nuclear 6, pseudogene):

AAACTCAAGACAATGGTGATAATGGTTTCTTTCAGGAGACCCAGGAGGGACTACCTTTCTGCGTATTCCTTTCTGTTCTTTAAAAATGTTAAACCATGGGGTGCTCGCTTCGGCAGCACATATACTAAAATTGGAACGAT

ACAGAGAAGATTAGCATGGCCCCTGCGCAAGGATGACACGCAAATTCGTGAAGCGTTCCATATTTTTATGCCGCATATCTACAACTATCTGATCTTTGACAAACCTGAGAAAAACAAGCAATGGGGAAAGGATTCCCTAT

TTAATAAATGGTGCTGGGAAAACTGGC

### Statistical analysis

For the evaluation of individual results of patients suffering from pleural plaques or asbestosis, delta CT values were compared to the 90% confidence interval of delta CT values of the healthy control group who were not exposed to asbestos. Values of diseased patients below or above the range of the 90% confidence interval of the healthy control group were considered up- or down-regulated.

Statistical analyses and the generation of graphs were carried out using the statistics program SPSS (version 28.0; SPSS Institute Inc., Chicago, USA). The Kolmogorov–Smirnov test was applied to determine the normal distribution of delta CT values of the leukocyte analysis. Since real-time RT-PCR delta CT values of miR-143-3p, miR-145-5p, miR-146b-5p and miR-204-5p were normally distributed, one-way analysis of variance (one-way ANOVA) with Bonferroni correction was performed. Delta CT values of miR-32-5p and miR-451a did not meet the normal distribution. Therefore, the Kruskal–Wallis test was used.

Delta CT values of miR-145-5p and miR-451a in serum samples were normally distributed and evaluated by using one-way ANOVA with Bonferroni correction. For the comparative analysis of delta CT values of miR-145-5p and miR-451a between leukocytes and serum, which were not normally distributed, the Kruskal–Wallis test was applied.

A value of *p* ≤ 0.05 was considered to be statistically significant.

To evaluate the effect of statistically significant differences, effect sizes [partial eta-squared (*η*^2^_p_) and Cohen’s f] according to one-way ANOVA were determined. *η*^2^_p_ values < 0.06, 0.06–0.14 or > 0.14 and effect sizes according to Cohen’s f of 0.10, 0.25 or 0.4 indicate small, medium or large effects, respectively. Small, medium or large effect size for the evaluation of Kruskal–Wallis test (*r*) are indicated by 0.1 ≤ *r* < 0.3, 0.3 ≤ *r* < 0.5 or *r* > 0.5, respectively. Moreover, values of difference and the 95% confidence interval were calculated for differences between groups.

A receiver operating characteristic (ROC) curve was performed and the area under the curve (AUC) calculated to evaluate the ability to discriminate between patients suffering from pleural plaques and healthy control participants using miR-146b-5p expression.

The coefficient of determination (*R*^2^) was calculated to evaluate regression regarding miR-145-5p or miR-451a expression between leukocytes (dependent variable) and serum (independent variable) using linear regression analysis [scatter (XY) plots]. An *R*^2^ value of 0 indicates no correlation and an *R*^2^ of 1 absolute correlation.

Moreover, Pearson and partial correlation coefficients (*r*) were calculated to determine the correlation of miR-145-5p or miR-451a expression between leukocytes and serum of patients suffering from mild, moderate or severe pleural plaques or asbestosis. Correlation values of −1 or 1 indicate either negative or positive correlations, respectively. A value of 0 indicates no correlation.

## Results

Pre-screening of 84 microRNAs using the miScript miRNA PCR Array Human Fibrosis showed that miR-32-5p, miR-143-3p, miR-145-5p, miR-146b-5p, miR-204-5p and miR-451a were expressed (*n* = 2; patients 21 and 27 of Table 3). These two patients were affected by moderate or severe pleural plaques and selected for pre-screening analysis based on the assumption that effects on microRNA regulation would be more pronounced with increased severity of the disease. Based on these pre-screening results, microRNAs expressed in moderately or severely affected patients were analyzed in leukocytes and serum of all study participants. Results showed differences in microRNA expression between patients but also between leukocytes and serum.

**Table 3:**
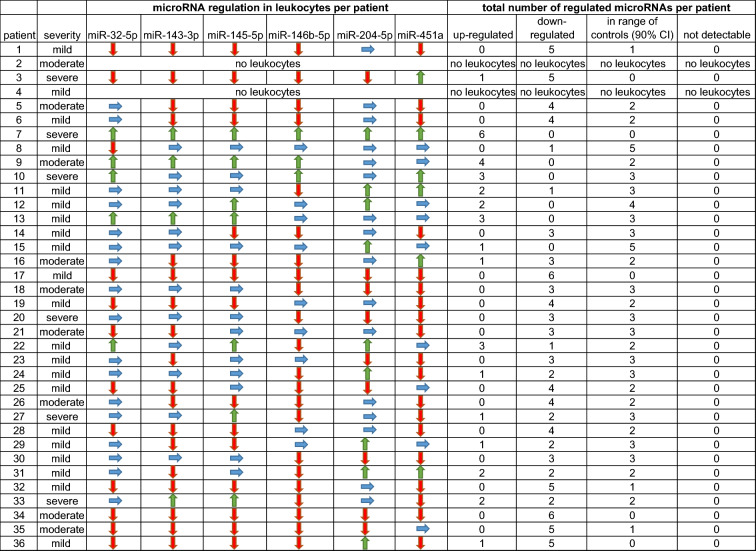
Individual microRNA regulation and number of microRNAs regulated in leukocytes per patient

: up-regulation, : down-regulation, : regulation in the range of controls,

*CI* confidence interval

### MicroRNA expression in leukocytes

The microRNAs miR-32-5p, miR-143-3p, miR-145-5p, miR-146b-5p, miR-204-5p and miR-451a, known to be involved in fibrosis, were detected in leukocytes of patients suffering from pleural plaques or asbestosis as well as in the healthy control group.

**Fig. 1:**
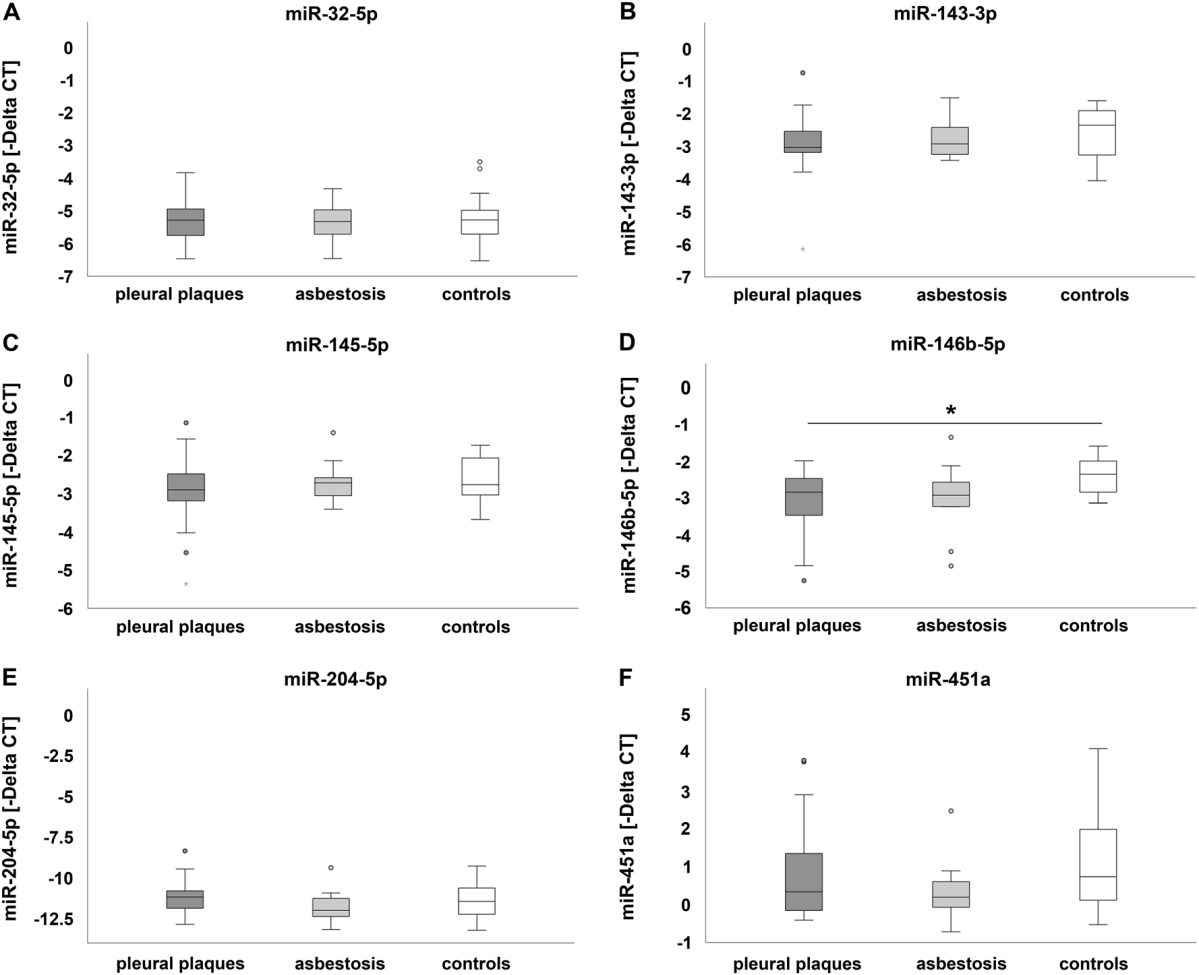
MicroRNA expression in leukocytes: comparison of miR-32-5p (**A**), miR-143-3p (**B**), miR-145-5p (**C**), miR-146b-5p (**D**), miR-204-5p (**E**) and miR-451a (**F**) expression in patients affected by pleural plaques or asbestosis and controls. Dark grey, light grey and white circles indicate outliers. The black asterisk (*) illustrates statistically significant differences with a likelihood of *p* ≤ 0.05

Delta CT values of miR-146b-5p in leukocytes were significantly down-regulated in patients affected by pleural plaques, but not in patients affected by asbestosis when compared to controls (Fig. 1D). The effect size calculation for miR-146b-5p down-regulation resulted in *η*^2^_p_ = 0.150 and *f* = 0.42, indicating a large effect. The value of difference was 0.725 and the 95% confidence interval was 0.070–1.381 when comparing healthy controls to patients suffering from pleural plaques. No significant differences in microRNA expression were detected comparing patients affected by pleural plaques to patients suffering from asbestosis. Expression of miR-32-5p, miR-143-3p, miR-145-5p, miR-204-5p and miR-451a showed slight differences between patients affected by pleural plaques or asbestosis as well as in controls. However, differences were not statistically significant between all three groups (Fig. 1A–C and E–F).

**Fig. 2:**
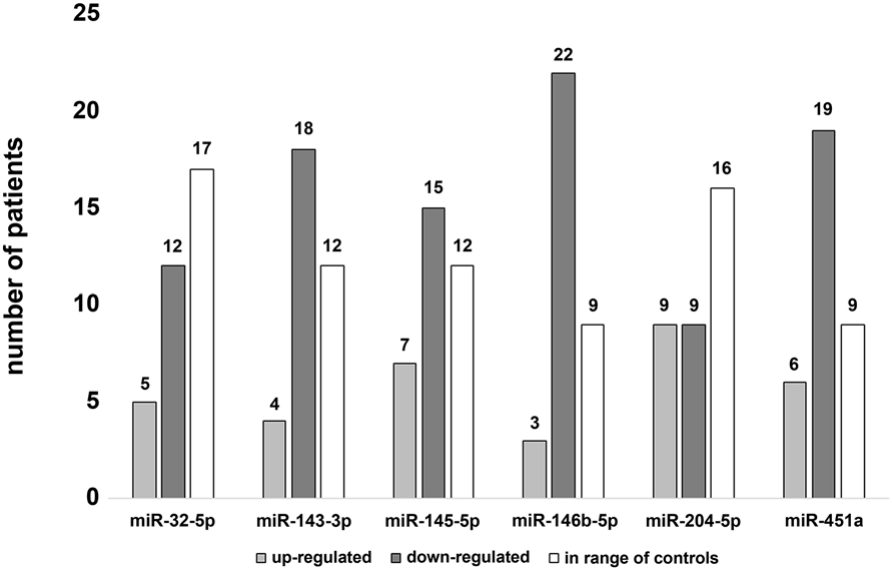
Number of patients affected by pleural plaques or asbestosis and the respective regulation of miR-32-5p, miR-143-3p, miR-145-5p, miR-146b-5p, miR-204-5p and miR-451a in leukocytes

Figure 2 displays in how many patients each analyzed microRNA was either up- or down-regulated or else in the range of the 90% confidence interval of controls in leukocytes. All six microRNAs analyzed were detectable. In most patients affected by the disease, these microRNAs were down-regulated. Several patients showed microRNA expression in the range of healthy non-exposed control individuals (Fig. 2).

Table 3 shows the individual variability of microRNA expression, summarized as frequency of up- or down-regulated microRNAs or unchanged expression compared to controls in leukocytes of each patient. In contrast to the majority of patients in whom microRNAs were down-regulated, in six patients most microRNAs analyzed were up-regulated (Table 3).

**Fig. 3:**
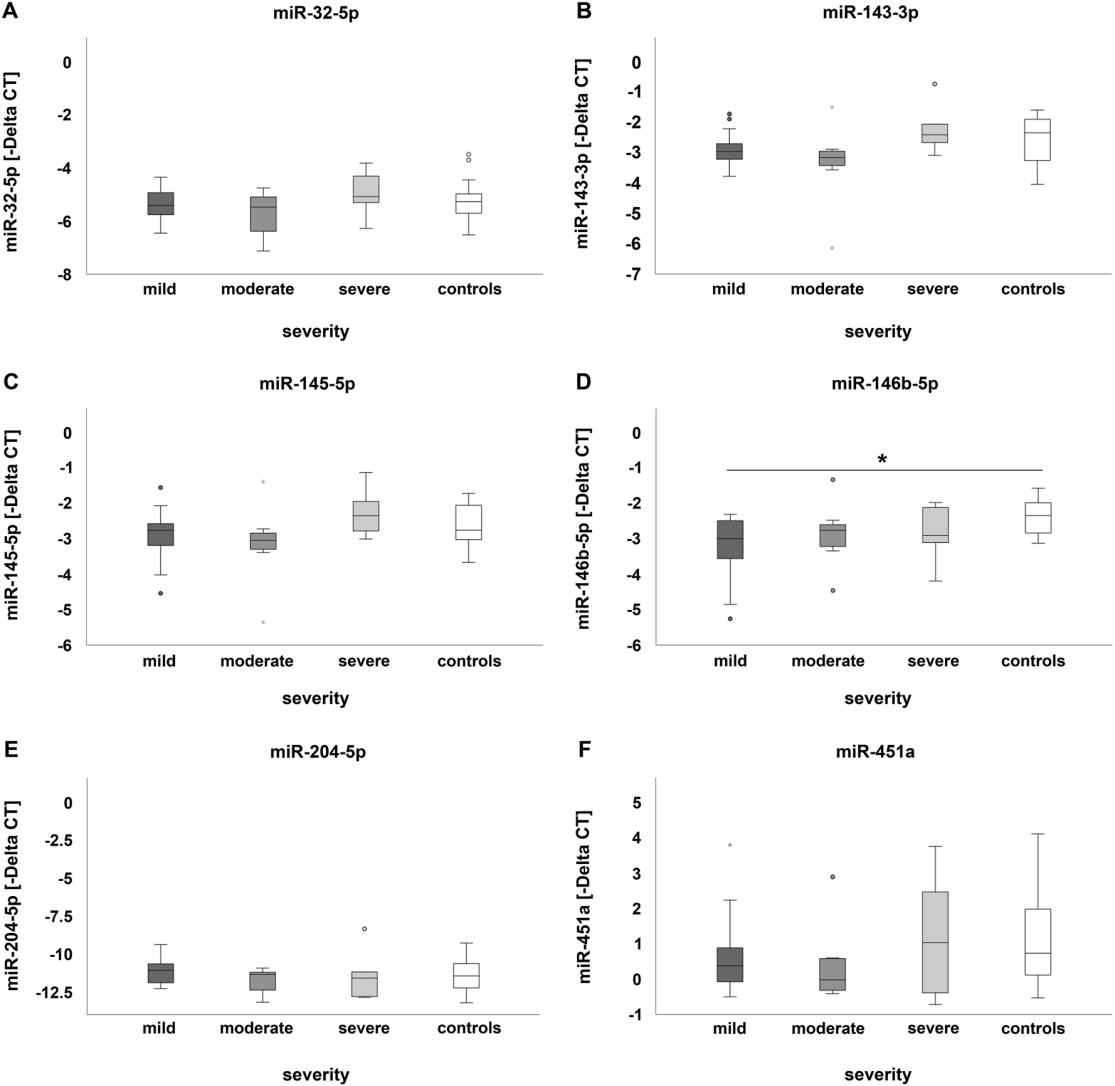
MicroRNA expression in leukocytes in relation to severity of disease: comparison of miR-32-5p (**A**), miR-143-3p (**B**), miR-145-5p (**C**), miR-146b-5p (**D**), miR-204-5p (**E**) and miR-451a (**F**) of patients affected by mild, moderate or severe pleural plaques or asbestosis as well as healthy controls. Dark grey, light grey and white circles indicate outliers. The black asterisk (*) illustrates statistically significant differences with a likelihood of *p* ≤ 0.05

To compare microRNA expression in respect to mild, moderate or severe disease, patients suffering from pleural plaques or asbestosis were pooled in the respective group. MicroRNA miR-146b-5p was significantly down-regulated in mildly diseased patients compared to the control group (Fig. 3D). Results of the effect size calculation for miR-146b-5p down-regulation indicated a large effect with *η*^2^_p_=0.178 and *f*=0.465. The value of difference was 0.848 and the 95% confidence interval 0.097–1.599 when comparing healthy controls to mildly diseased patients. No statistically significant differences were detectable when comparing miR-146b-5p expression of mildly diseased patients to that of moderately or severely diseased patients (Fig. 3D). Moreover, expression of microRNAs miR-32-5p, miR-143-3p, miR-145-5p, miR-204-5p and miR-451a did not differ in regard to the extent of pleural plaques or severity of asbestosis (Fig. 3A–C and E–F).

### Receiver operating characteristic (ROC) curve analysis

To evaluate discrimination ability between patients suffering from pleural plaques and healthy controls using miR-146b-5p, ROC curve analysis was performed and the area under the ROC curve (AUC) was determined. ROC curve and area under the curve (AUC) allow discrimination between diseased and healthy patients. Generally, a ROC curve located closer to the upper left-hand corner of the image indicates greater discrimination ability between diseased and healthy individuals. The area under the curve (AUC) summarizes the overall accuracy of the test and ranges from 0 to 1 (two-dimensionally). A value of 0 indicates low accuracy and a value of 1 indicates high accuracy, the latter correlates to a high ability to differentiate between diseased and healthy individuals. An AUC of 0.5 suggests no discrimination. An AUC of 0.7–0.8 is considered acceptable, 0.8–0.9 is considered excellent and above 0.9 is considered outstanding [50].

**Fig. 4:**
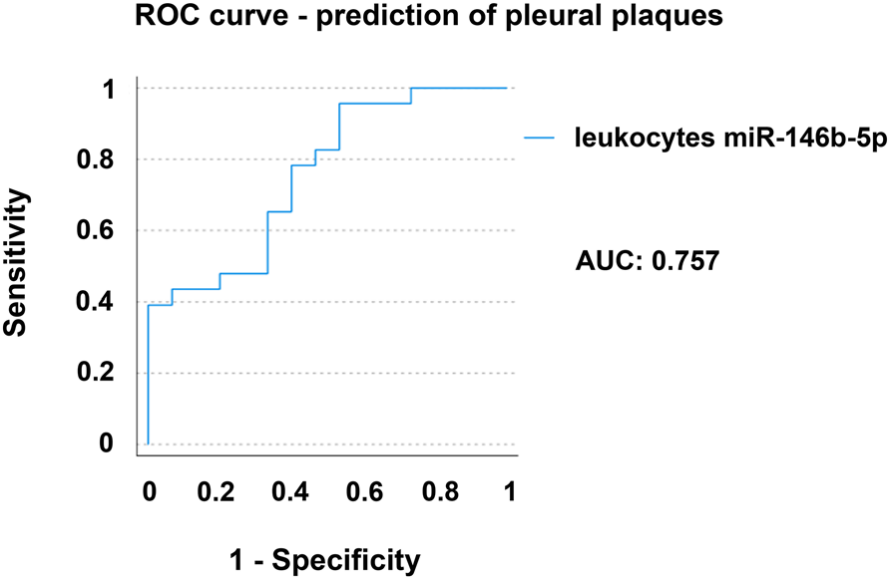
ROC curve and AUC of miR-146b-5p depicting acceptable ability to discriminate between patients suffering from pleural plaques and healthy controls

In the underlying study an AUC of 0.757 for miR-146b-5p expression in leukocytes indicated an acceptable ability to discriminate between patients suffering from pleural plaques and healthy controls (Fig. 4).

### MicroRNA expression in serum

**Fig. 5:**
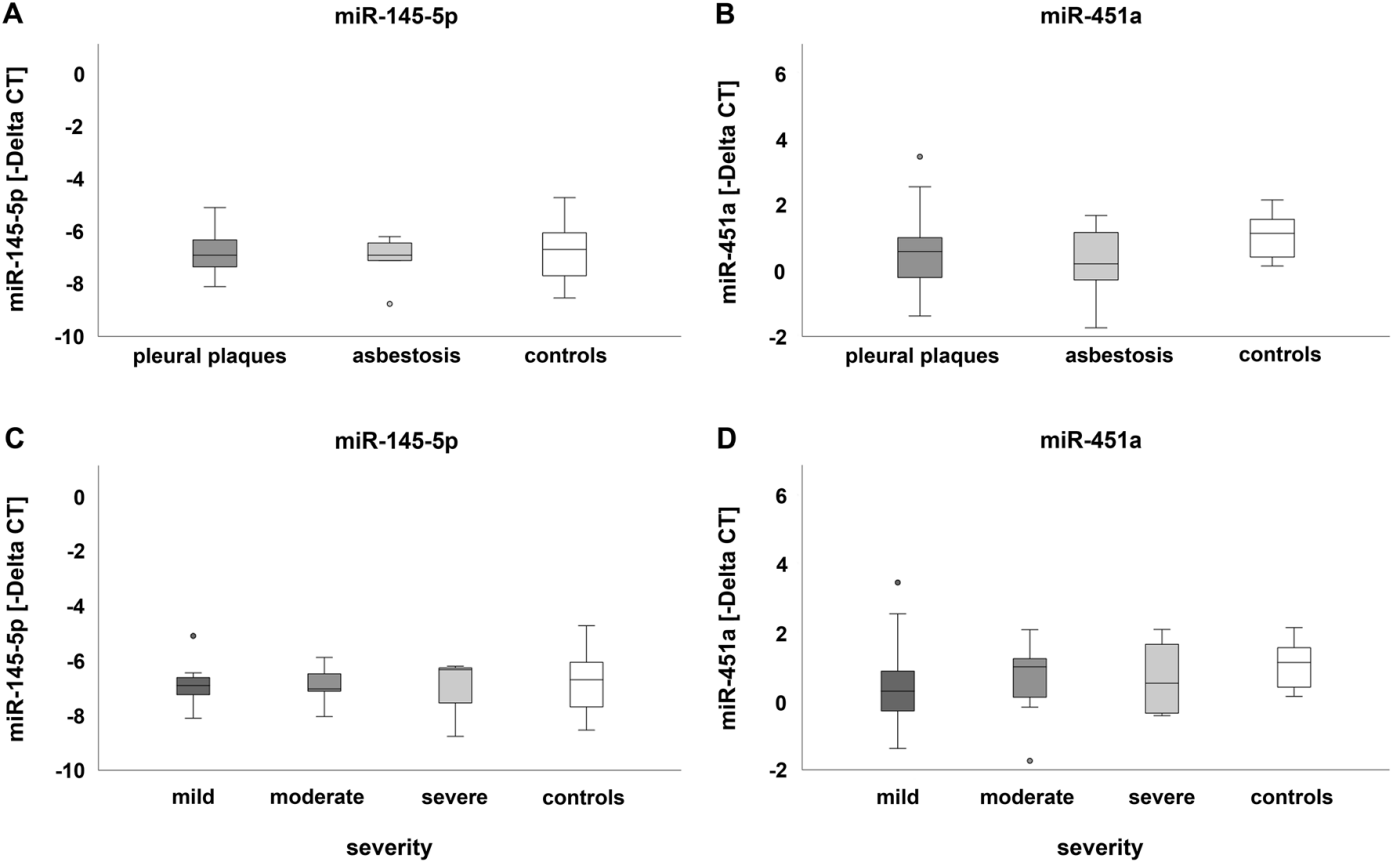
MicroRNA expression in serum: comparison of miR-145-5p (**A**) and miR-451a (**B**) expression in patients affected by pleural plaques or asbestosis and controls as well as of miR-145-5p (**C**) and miR-451a (**D**) with regard to severity of the disease. Dark- and light-grey circles indicate outliers

MicroRNAs miR-32-5p, miR-143-3p, miR-146b-5p and miR-204-5p were neither detectable in serum samples of patients affected by pleural plaques or asbestosis nor in the control group, but microRNA miR-451a was detectable in all serum samples analyzed. In contrast, miR-145-5p was expressed only in 10 of 26 patients exhibiting pleural plaques and in 5 of 10 asbestosis patients as well as in 10 of 15 study participants in the control group. However, microRNAs were not significantly (up- or down)-regulated in serum samples of either group, independent from the degree of severity of the disease (Fig. 5).

**Fig. 6:**
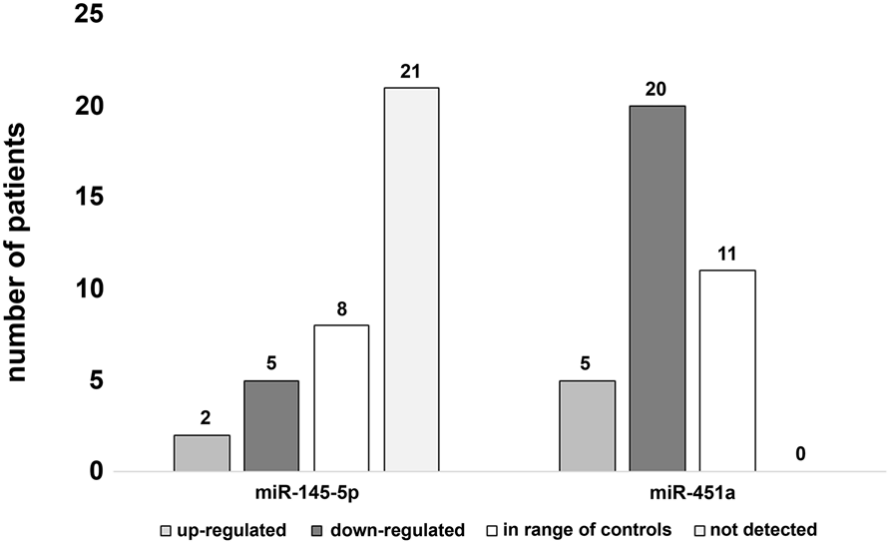
Number of patients affected by pleural plaques or asbestosis and the respective regulation of miR-145-5p and miR-451a in serum

Still, considering the number of patients, in serum of most patients, the expression of miR-451a was down-regulated or in the range of controls as shown in Fig. 6. Nevertheless, expression levels were similar to those of the control group, resulting in no significant differences. In serum of most patients, miR-145-5p was not detected or in the range of healthy non-exposed controls (Fig. 6).

Individual microRNA regulation profiles are presented in Table 4. It shows in how many patients each analyzed microRNA was up- or down-regulated or in the range of the 90% confidence interval of the controls in serum. In most patients, microRNAs analyzed were down-regulated or not detectable.

**Table 4:**
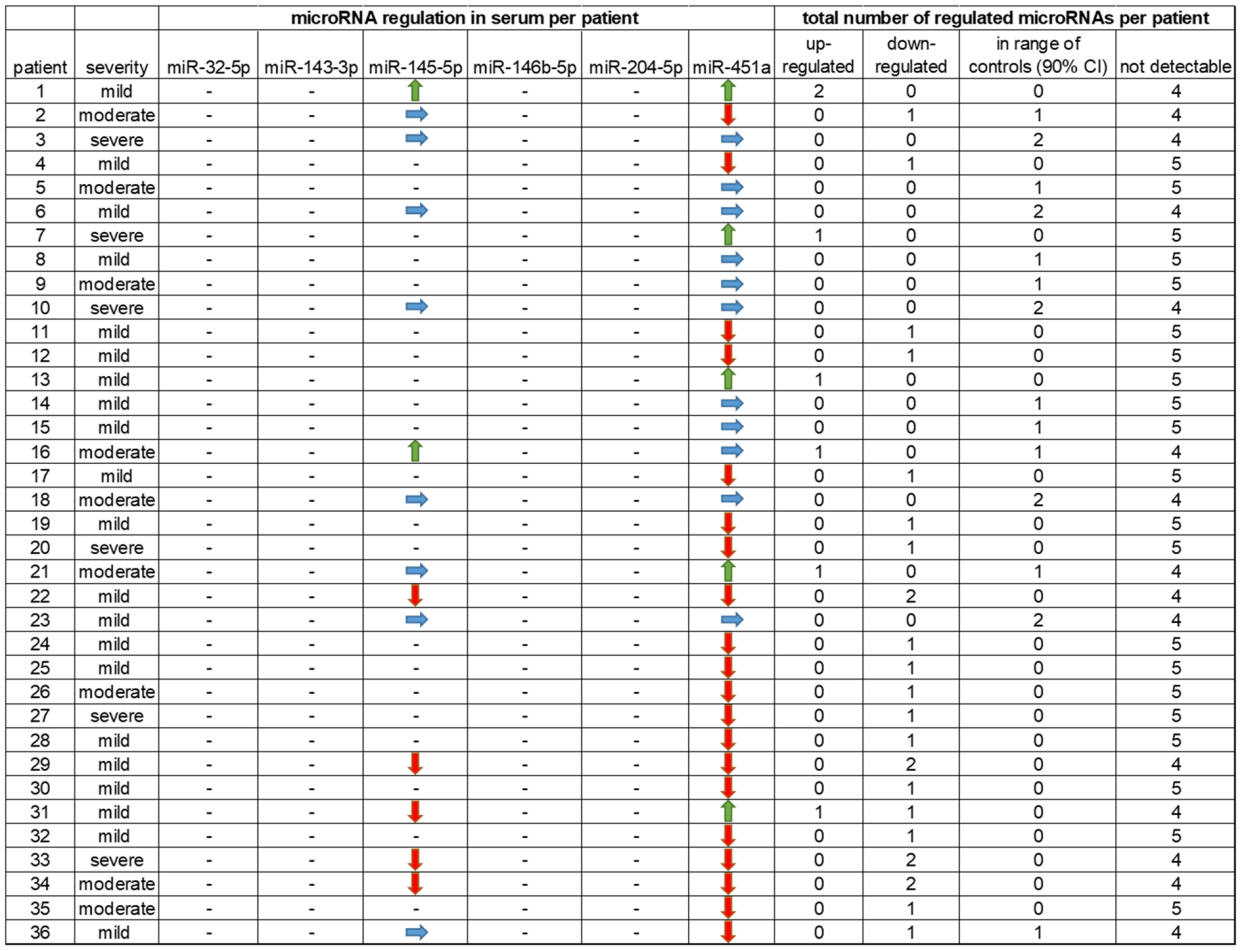
Individual microRNA regulation and number of microRNAs regulated in serum per patient

: up-regulation, : down-regulation, : regulation in the range of controls,

– no regulation, *CI* confidence interval

### Comparison of microRNA expression in leukocytes and serum

**Fig. 7:**
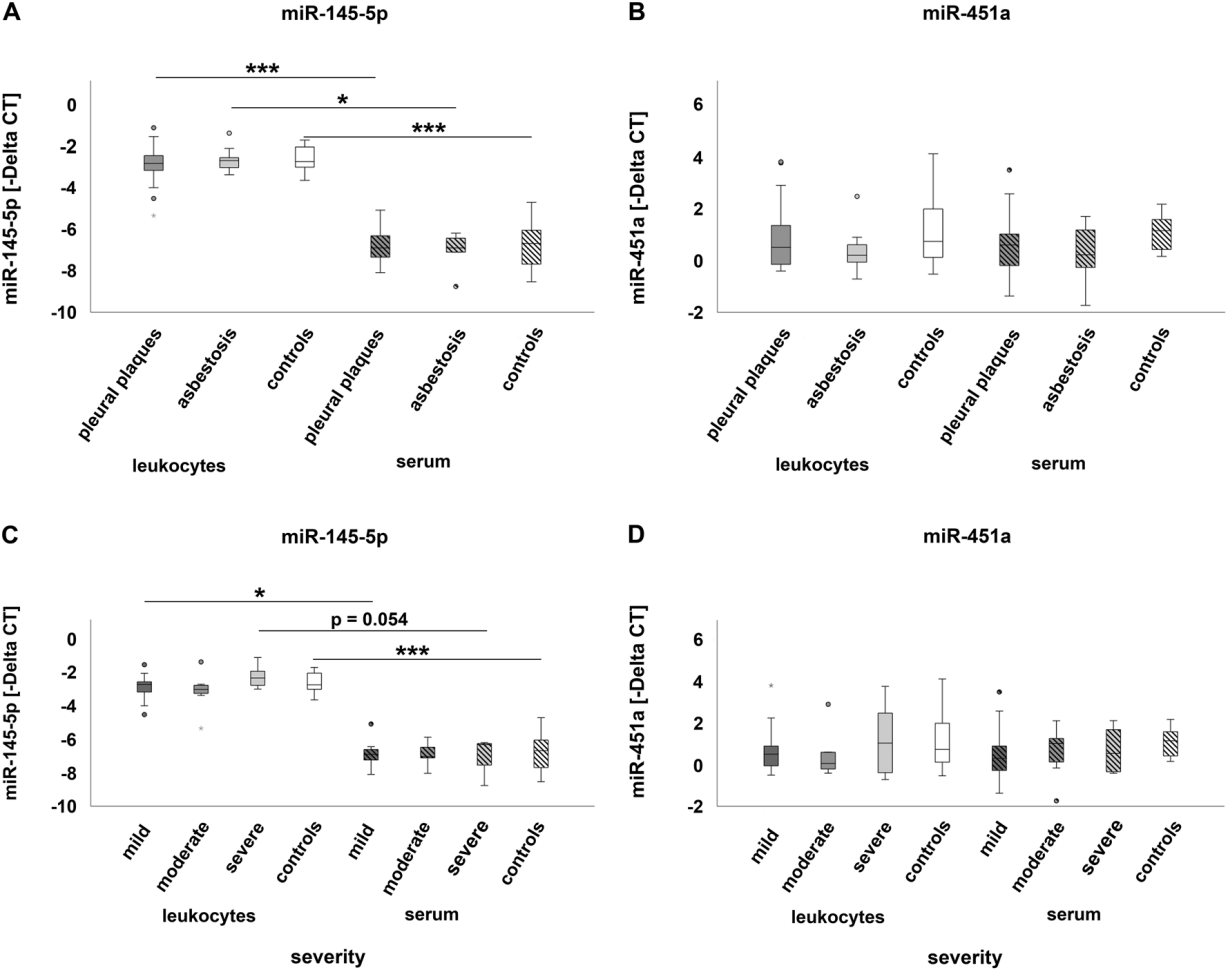
MicroRNA expression in leukocytes and serum: comparison of miR-145-5p (**A**) and miR-451a (**B**) expression in patients affected by pleural plaques or asbestosis and controls as well as of miR-145-5p (**C**) and miR-451a (**D**) with regard to severity of the disease. The dark grey and light grey circles indicate outliers. The black asterisks * and *** illustrate statistically significant differences with a likelihood of *p* ≤ 0.05 or p ≤ 0.001, respectively

The comparison of microRNA expression between leukocytes and serum showed significant differences. Not all microRNAs evidenced in leukocytes were detectable in serum samples. Only miR-145-5p and miR-451a were expressed in leukocytes and serum. Therefore, a comparison of expression differences between leukocytes and serum was performed for these two microRNAs exclusively (Fig. 7).

MicroRNA miR-145-5p was significantly down-regulated in serum compared to leukocytes of patients suffering from pleural plaques or asbestosis (Fig. 7A), independent from the severity of the disease (Fig. 7C) as well as in controls. Effect size (*r*) calculation according to the Kruskal–Wallis test for miR-145-5p down-regulation when comparing serum to leukocytes of patients suffering from pleural plaques, asbestosis or controls resulted in values of *r* = 0.38, 0.50 or 0.51, respectively, and indicated a medium effect. Considering disease severity, effect size for miR-145-5p down-regulation between serum and leukocytes in mildly diseased patients was *r* = 0.43 and indicated a medium effect as well. Neither pleural plaques nor asbestosis or degree of severity did affect the expression of miR-451a (Fig. 7B and D).

**Fig. 8:**
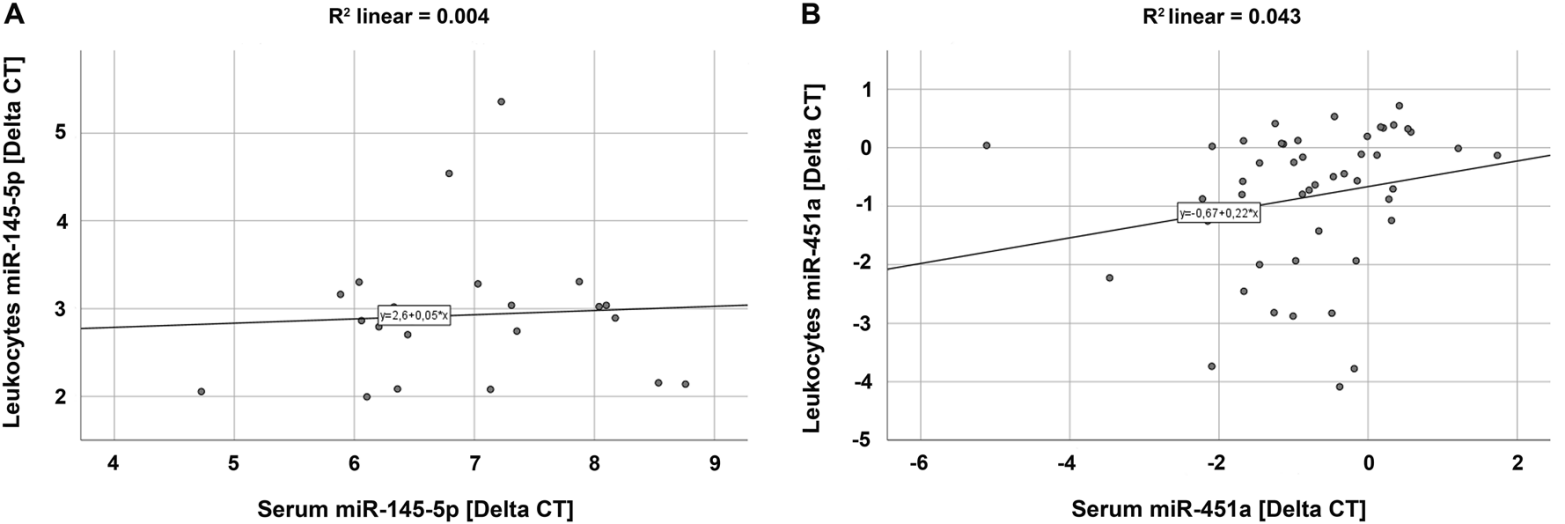
Scatter (XY) plots depicting no correlation of miR-145-5p (**A**) and miR-451a (**B**) expression between leukocytes and serum in patients suffering from pleural plaques or asbestosis as well as healthy control participants

Regression analysis revealed an *R*^2^ value of 0.004 for miR-145-5p and an *R*^2^ of 0.043 for miR-451a, indicating no correlation between leukocytes and serum (Fig. 8).

The Pearson correlation (*r*) supported the result of the *R*^2^ analysis and revealed values of *r* = 0.061 and *r* = 0.208 for miR-145-5p and miR-451a, respectively, indicating no correlation as well.

Partial correlation in leukocytes and serum showed a value of −0.02 for miR-145-5p and of 0.159 for miR-451a when controlling for pleural plaques or asbestosis. When controlling for disease severity, partial correlation revealed a value of −0.009 for miR-145-5p and 0.166 for miR-451a. Thus, regarding miR-145-5p and miR-451a expression, results of the partial correlation analysis confirmed no correlation between leukocytes and serum when controlling for pleural plaques, asbestosis or disease severity, accordingly.

## Discussion

Asbestos-related pleural plaques and asbestosis are fibrotic changes of pleura and/or lung caused by asbestos [6, 51]. MicroRNAs miR-32-5p, miR-143-3p, miR-145-5p, miR-146b-5p, miR-204-5p and miR-451a are involved in fibrosis [37–42] and are regulated in mesothelioma or lung cancer [36, 43–48]. Although asbestos-related pleural plaques or asbestosis do not inevitably lead to cancer, patients are at higher risk of developing mesothelioma or lung cancer [12, 52, 53]. Several studies have shown that microRNA down-regulation is associated with mesothelioma and lung cancer [36, 43–45], but less is known in this relation to asbestosis [54].

Identification of microRNAs expressed in patients suffering from asbestos-related pleural plaques or asbestosis might contribute to a risk assessment of developing mesothelioma or lung cancer.

Since blood sampling is a routine diagnostic method and less invasive compared to taking biopsies, circulating microRNAs may assist in the evaluation of disease progression and cancer risk assessment, especially in occupational diagnostic approaches.

Thus, the aim of this study was to identify microRNAs expressed in leukocytes and/or serum of patients affected by asbestos-related pleural plaques or asbestosis that could be suitable as biomarkers for disease progression or severity and cancer risk assessment.

### MicroRNA expression in leukocytes

In the present study, microRNA miR-146b-5p was significantly down-regulated in leukocytes of patients exhibiting pleural plaques. The majority of patients in this study showed a radiological mild extent of pleural plaques. However, in patients affected by asbestosis, no significant differences were detected in microRNA expression.

Pleural plaques and asbestosis are progressive diseases [53, 55]. Progression of fibrosis can change the regulation of microRNAs [56]. Interestingly, with regard to severity, miR-146b-5p was down-regulated only in patients impaired by mild pleural plaques or mild asbestosis. However, most asbestosis patients showed moderate or severe changes of pleura and lung (7 out of 10). Considering moderately or severely affected patients, miR-146b-5p was neither up- nor down-regulated in leukocytes.

Weber et al*.* (2017) detected no significant differences in the regulation of miR-146b-5p when comparing plasma samples of mesothelioma patients with plasma samples of asbestos-exposed control participants [57]. This result is in accordance with our finding in leukocytes, which showed no significant differences in miR-146b-5p regulation when comparing patients affected by moderately and severely asbestos-related pleural plaques or asbestosis as well as non-exposed healthy controls. Accordingly, down-regulation of miR-146b-5p in leukocytes could be interpreted as an indicator for mild asbestos-related diseases.

Interestingly, microRNA miR-146b-5p was down-regulated in patients suffering from pleural plaques, but not from asbestosis. In patients suffering from asbestosis the lung parenchyma is fibrotic, mostly accompanied by pleural plaques [58]. Lung parenchyma differs from tissue of the pleura. Whereas lung parenchyma consists of epithelial cells [59], pleura tissue comprises mesothelial cells [60]. Presumably, different cell types might display different microRNA expression patterns, which could explain that miR-146b-5p was significantly down-regulated only in patients suffering from pleural plaques, but not in asbestosis patients. Although pleural plaques were present in asbestosis patients in this study as well, the lung parenchyma is proportionally larger than the pleural tissue. Thus, effects of pleural plaques on microRNA regulation in leukocytes might have been less pronounced in asbestosis patients.

Leukocytes are of mesenchymal origin, like mesothelial cells of the pleura. Presumably, the down-regulation of miR-146b-5p only in leukocytes of patients suffering from asbestos-related pleural plaques could suggest a correlation between microRNA regulation in leukocytes and pleural plaques manifestation.

Our study revealed no statistically significant differences in expression of miR-32-5p, miR-143-3p, miR-145-5p, miR-204-5p and miR-451a in leukocytes of patients exhibiting mild, moderate or severe extent of pleural plaques or asbestosis. Nevertheless, looking at patients individually, these microRNAs were predominantly down-regulated or in the range of controls who were not exposed to asbestos.

However, in a small group of patients (6 of 34), most microRNAs were up-regulated although no explanation could be found for that finding in relation to anamnesis, disease severity, hemogram and biochemical parameters. The same parameters did not differ noticeably between patients with up- or down-regulated microRNA expression.

The regulation of certain microRNAs can differ between younger and older individuals [61]. Although age itself can influence the microRNA regulation, to the best of our knowledge, the regulation of miR-146b-5p has not been shown to be affected by increased age. Given that we did not detect significant differences in the regulation of miR-32-5p, miR-143-3p, miR-145-5p, miR-204-5p and miR-451a, we conclude that the age difference between diseased patients and healthy control participants did not influence the regulation of microRNAs analyzed in the underlying study.

### MicroRNA expression in serum

In serum samples of patients affected by pleural plaques or asbestosis and control participants, miR-32-5p, miR-143-3p, miR-146b-5p and miR-204-5p were either not traceable or their expression was at a low, not evaluable detection level (CT value > 35). Therefore, they do not seem suitable for evaluating the progression of asbestos-related pleural plaques or asbestosis in serum samples.

Although all microRNAs analyzed were shown to be involved in fibrosis, in this study only miR-145-5p and miR-451a were detectable in serum samples of patients affected by asbestos-related pleural plaques or asbestosis as well as in control individuals who were not exposed to asbestos. This indicates that these microRNAs are physiologically expressed in serum and that microRNA expression in serum of patients suffering from pleural plaques or asbestosis did not differ from controls.

Damaged tissue can release microRNAs into serum [28] and there is generally a positive correlation between the quantity of microRNAs that are released and damage severity [62]. Thus, since the majority of patients in this study were only mildly affected by asbestos-related pleural plaques, less microRNAs might have been released into serum. This could explain the similar expression of miR-145-5p and miR-451a between diseased patients and healthy control individuals. Unexpectedly, in serum of patients suffering from moderate or severe asbestosis, we detected no significant differences in microRNA expression compared to non-exposed healthy controls, contradicting the positive correlation between the quantity of microRNAs that are released and damage severity. Unlike in leukocytes this indicates that disease severity did not influence microRNA expression in serum of patients suffering from asbestos-related pleural plaques or asbestosis.

### Comparison of microRNA expression in leukocytes and serum

Our study revealed differentially expressed microRNAs in leukocytes and serum. MicroRNA miR-146b-5p was significantly down-regulated in leukocytes but not detected in serum. Interestingly, miR-145-5p was significantly differently regulated in leukocytes and serum. MicroRNA miR-145-5p expression was higher in leukocytes than in serum.

Pascut et al*.* (2019) suggested that expression of microRNAs in serum could be affected by the disease and not by leukocytes [24], since tissue can release microRNAs into serum. Thus, leukocytes or serum could be analyzed independently to obtain information about the progression of the disease. Considering a correlation between higher microRNA levels in serum and increased severity of tissue damage, serum might support detection of moderate or severe asbestos-related pleural plaques or asbestosis. However, even more severely affected patients than the individuals who participated in this study were not available. Presumably, microRNA analysis in serum would be more suitable to evaluate disease progression in mesothelioma or asbestos-related lung cancer. Thus, since we could not detect significant differences in microRNA expression in serum of mildly, moderately or severely diseased patients, leukocytes seem more suitable for the microRNA analysis to evaluate progression of asbestos-related pleural plaques or asbestosis and a possible cancer risk.

MicroRNA miR-451a was equally regulated when comparing leukocytes and serum of patients affected by pleural plaques or asbestosis, and this was independent of the level of severity.

One might consider the rather small sample size of nine moderately and six severely diseased patients as well as the relatively large age gap between patients and control participants as limitations in this study. However, since asbestos-related diseases show long latency, patients usually are of advanced age. The older individuals who presented to our clinic showed comorbidities, so the control group consisted of comparably young and healthy individuals. However, the respective results that showed no significant differences when comparing older diseased participants with younger healthy control individuals suggest that age did not affect microRNA regulation in the underlying study.

## Conclusion

MicroRNA miR-146b-5p was down-regulated in leukocytes of patients who suffered from pleural plaques. Considering disease severity, miR-146b-5p was significantly down-regulated in leukocytes of mildly diseased patients.

Since in serum no significant changes in microRNA expression were detectable, leukocytes seem more suitable for disease and potential cancer risk assessment in patients suffering from asbestos-related pleural plaques or asbestosis.

Follow-up examination of patients who showed miR-146b-5p down-regulation in leukocytes needs to be undertaken to determine whether this microRNA might be an early indicator and potentially biomarker for an increased cancer risk in patients suffering from asbestos-related diseases.

## Data Availability

All data generated or analyzed during this study are included in this published article. The datasets used and/or analyzed during the current study are available from the corresponding author on reasonable request.

## Abbreviations

CI: Confidence interval
CT: Cycle threshold
EDTA: Ethylenediaminetetraacetic acid
hsa: *Homo sapiens*
ICD-10: International Statistical Classification of Diseases and Related Health Problems
ICOERD: International Classification of Occupational and Environmental Respiratory Diseases
ILO: International Classification of Radiographs of Pneumoconiosis of the International Labour Organization
mRNA: Messenger RNA
miR/miRNA: MicroRNA
RNA: Ribonucleic acid
RT-PCR: Reverse transcription polymerase chain reaction
